# Allogeneic hematopoietic stem cell transplantation in Hodgkin lymphoma in Switzerland, 20 years of experience: 2001–2020

**DOI:** 10.1002/jha2.629

**Published:** 2022-12-25

**Authors:** Helena Simeunovic, Martina Dickenmann, Mitja Nabergoj, Helen Baldomero, Stavroula Masouridi‐Levrat, Gayathri Nair, Urs Schanz, Jacob Passweg, Alicia Rovo, Yves Chalandon, Ekaterina Rebmann

**Affiliations:** ^1^ University Hospital of Bern (Inselspital) Bern Switzerland; ^2^ Aarau Kantonsspital Aarau Switzerland; ^3^ Hôpital Riviera‐Chablais Vaud‐Valais, Rennaz Switzerland; ^4^ Univeristy Hospital of Basel (USB) Basel Switzerland; ^5^ Department of Oncology Division of Hematology Geneva University Hospitals (HUG) Geneva Switzerland; ^6^ University Hospital of Zurich (USZ) Zurich Switzerland; ^7^ Faculty of Medicine University of Geneva Geneva Switzerland; ^8^ Hospital of Neuchâtel (RHNE) Nuechatel Switzerland

**Keywords:** allogeneic stem cell transplantation, Hodgkin lymphoma

## Abstract

Despite the high cure rate with initial therapy, approximately 10% of Hodgkin lymphoma (HL) patients are refractory to initial treatment, and up to 30% of patients will relapse after achieving initial complete remission. Despite promising initial results of treatment by immune checkpoint inhibitors, most patients will eventually progress. We analyzed 62 adult patients with relapsed or refractory HL (rrHL) treated by allogeneic hematopoietic stem cell transplantation (allo‐HSCT) in one of three University Hospitals of Switzerland (Zurich, Basel, and Geneva) between May 2001 and January 2020. The primary endpoint was overall survival (OS). Secondary endpoints were relapse‐free survival (RFS), non‐relapse mortality (NRM), and relapse incidence, which were assessed in univariate analysis. The median follow‐up was 61 months (interquartile range 59–139). The 2‐ and 5‐year OS was 54% (standard error (SE) ±12) and 50.2% (SE ±13.3), respectively, and the 2‐ and 5‐year RFS was 40.7% (SE ±16.3) and 34.4% (SE ±19.0), respectively. NRM was 23.1% (SE ±2.2) and 27.4% (SE ±2.5) at 2 and 5 years, respectively. The cumulative incidence of relapse was 36.1% (SE ±5.6) at 2 years and 38.2% (SE ±6.6) at 5 years. Our analysis of allo‐HSCT outcomes in the context of rrHL shows encouraging OS and RFS rates, with the mortality rate reaching plateau at 50% at 2 years after allo‐HSCT. This confirms that allo‐HSCT still remains as a potentially curative option for half of patients with rrHL.

## 1 INTRODUCTION

Despite the high cure rate with frontline therapy, up to 30% of Hodgkin lymphoma (HL) patients will relapse after achieving complete remission (CR) or will develop primary refractory disease (rrHL) [1, 2]. Targeted therapies such as checkpoint inhibitors (CPI) and anti‐CD30 monoclonal antibodies (brentuximab vedotin, BV) yield promising results with high response rates in rrHL [3]. However, most patients with rrHL would eventually relapse or progress [2]. For eligible rrHL patients, allogeneic hematopoietic stem cell transplantation (allo‐HSCT) currently remains the last potentially curative option, leading to sustainable remission in approximately 50% of these patients [2]. The role of allo‐HSCT in rrHL patients has been controversial due to the high rates of non‐relapse mortality (NRM) [4]. However, allo‐HSCT represents the potentially curative treatment in this setting [1].

Here, we report a retrospective, multicenter, registry‐based analysis (Swiss Blood Stem Cell Transplantation and Cellular Therapy, SBST) of the Swiss experience in allo‐HSCT for HL from 2001 to 2020.

## 2 MATERIALS AND METHODS

We retrospectively collected the data from 62 patients with HL treated with allo‐HSCT in three University Hospitals of Switzerland (Zurich, Basel, and Geneva) between May 2001 and January 2020. The primary endpoint was overall survival (OS). Secondary endpoints were relapse‐free survival (RFS), NRM, and relapse incidence (RI), which were assessed by univariable analysis.

## 3 STATISTICAL ANALYSIS

Median values and interquartile ranges (IQR, Q1–Q3) were used for continuous variables and compared using Wilcoxon signed‐rank test; categorical variables were summarized as counts and percentages and compared using the chi‐squared or Fisher's exact test. Median follow‐up was calculated as per the reverse Kaplan–Meier method. OS and RFS were assessed using the Kaplan–Meier method. Competing risk outcomes (NRM and RI) were assessed using cumulative incidence to accommodate competing risks, with only death considered as a competing event among analyzed outcomes. All statistical tests were two‐sided, and *p* < 0.05 was considered significant. Analyses were performed using R RStudio (version 1.4.1106; R Foundation, USA) using the packages survival (version 3.2.7), cmprsk (version 2.2.10), and prodlim (version 2019.11.13).

## 4 RESULTS

The median follow‐up was 87 months (IQR 59–139). The median age of patients at the time of allo‐HSCT was 28 years (24–33), and there were more male patients (46, 74%). Prior autologous HSCT was performed in 51 (82%) patients, and eight (13%) patients had previously undergone allo‐HSCT. Performance status with Karnofsky index ≥80 was reported in 42 (98%) patients. Only 18 (31%) of 62 patients were in CR at the time of allo‐HSCT.

Regarding conditioning regimen, 48 (77%) patients were treated with a reduced‐intensity conditioning (RIC) regimen and 13 (21%) received total body irradiation. Stem cell source was peripheral blood for 54 (87%) patients, and eight (13%) patients received bone marrow. The donor was human leukocyte antigen (HLA)‐identical sibling or an HLA‐matched unrelated donor for 29 (47%) and 22 (35%) patients, respectively, three (5%) patients had a mismatched unrelated donor and eight (13%) patients had a partially matched related donor. Successful neutrophil engraftment occurred in 60 (98%) patients in a median of 15 days (13–17).

**FIGURE 1:**
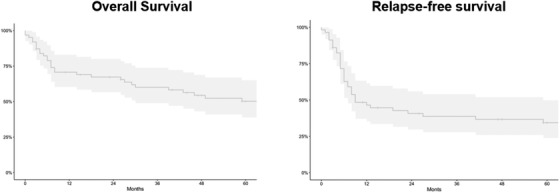
Five‐year overall survival and relapse‐free survival

The 2‐ and 5‐year OS was 54% (SE ±12) and 50.2% (SE ±13.3), respectively, and the 2‐ and 5‐year RFS was 40.7% (±16.3) and 34.4% (SE ±19.0), respectively. NRM was 23.1% (SE ±2.2) and 27.4% (SE ±2.5) at 2 and 5 years, respectively. The cumulative incidence of relapse was 36.1% (SE ±5.6) at 2 years and 38.2% (SE ±6.6) at 5 years (Table 1 and Figure 1).

**TABLE 1:** Patient's clinical characteristics

	Total (62)	Missing
Center, *n* (%)		0
HUG	22 (35)	
USB	21 (34)	
USZ	19 (31)	
Median follow‐up (KM‐1) (months) (IQR)	87 (59–139)	
Age at allo‐SCT (years), median (IQR)	28 (24–33)	
<28 years, *n* (%)	27 (44)	
≥28 years, *n* (%)	35 (56)	
Age at HL diagnosis (years), median (IQR)	24 (20–30)	
Year of allo‐SCT, median (IQR)	2010 (2006–2014)	
Previous stem cell transplantation, *n* (%)		1
None	3 (5)	
Auto	51 (82)	
Allo	8 (13)	
Sex, *n* (%)		0
Male	46 (74)	
Female	16 (6)	
Karnofsky, *n* (%)		19
<80	1 (2)	
≥80	42 (98)	
Disease status at allo‐SCT, *n* (%)		3
CR	18 (31)	
PR	12 (20)	
SD	1 (2)	
PD	5 (8)	
Primary refractory	3 (5)	
Relapse	20 (34)	
Stem cell source, *n* (%)		0
BMSC	8 (13)	
PBSC	54 (87)	
Ex vivo manipulation (T‐depletion), *n* (%)		1
No	50 (82)	
Yes	11 (18)	
Type of donor, *n* (%)		0
HLA‐identical sibling	29 (47)	
HLA‐matched unrelated donor	22 (35)	
MMUD	3 (5)	
PMRD	8 (13)	
TBI, *n* (%)		0
Yes	13 (21)	
No	49 (79)	
Conditioning regimen, *n* (%)		0
MAC	14 (23)	
RIC/other	48 (77)	
Donor age (years), median (IQR)	32 (26–40)	
Donor sex, *n* (%)		0
Male	41 (66)	
Female	21 (34)	
Engraftment, *n* (%)		1
Yes	60 (98)	
No	1 (2)	
Neutrophil engraftment (days), median (IQR)	15 (13–17)	

Abbreviations: allo‐SCT, allogeneic stem cell transplantation; BMSC, bone marrow stem cell; CR, complete remission; HL, Hodgkin lymphoma; HLA, human leukocyte antigen; HUG, Geneva University Hospitals; IQR, interquartile range; MAC, myeloablative conditioning; MMUD, mismatched unrelated donor; PBSC, peripheral blood stem cell; PMRD, partially mismatched related donor; PR, partial remission; SD, stable disease; PD, progressive disease; RIC, reduced‐intensity conditioning; TBI, total body irradiation; USB, University Hospital of Basel; USZ, University Hospital of Zurich.

## 5 DISCUSSION

First reports on the outcomes of allo‐HSCT in HL described quite discouraging results. In 1996, Gajewski et al. [5] reported a 3‐year probability of survival at around 21% and a 3‐year disease‐free survival rate as low as 15%. Since then, the introduction of RIC regimens, improvement of supportive care, and graft‐versus‐host disease (GvHD) prevention and treatment have contributed to better outcomes and lower NRM rates [6, 7, 8, 9].

As for other hematologic diseases, the application of allo‐HSCT in rrHL has been expanded in HL by alternative (haploidentical) donor's usage [10, 11]. This was possible because of constant improvement of peri‐transplant treatment, including lower toxicity by the usage of new agents such as BV and CPI and better GvHD control in early post‐transplant period by cyclophosphamide and antithymocyte globulin usage [10, 12, 13]. Because of the high efficacy, BV and CPI and their combinations are now under extensive investigation for optimal bridging strategies prior to allo‐SCT [13, 14, 15].

Thus, the constant improvement of peri‐transplant settings allows the patient to obtain higher survival results than previously reported [1]. A previous analysis of disease outcome of 709 HL patients treated by allo‐HSCT (EBMT register) published in 2020 showed a 3‐year OS as high as 77% and a 3‐year progression‐free survival of 63% [11].

Our analysis of allo‐HSCT outcomes in the context of rrHL also shows encouraging 5‐year OS and RFS rates, with the mortality rate reaching a plateau at 50% at 2 years after allo‐HSCT. Relatively high NRM rates in this young population could be explained by accumulated toxicities after multiple pre‐transplant treatment lines.

This is the first study of allo‐HSCT outcomes in patients with rrHL in Switzerland. The analysis is retrospective and register‐provided (SBST), so it lacks some important details, for example, regarding the number and type of pre‐transplant treatments as well as the full information about type and incidence of GvHD. We still believe that this brief survival extraction could be considered a very important message for allo‐HSCT in the setting of rrHL.

## 6 CONCLUSION

The results of this retrospective study suggest that allo‐HSCT may be a viable treatment option for the difficult‐to‐treat population of rrHL.

## AUTHOR CONTRIBUTIONS

*Data collection, interpretation, and manuscript writing*: Helena Simeunovic. *Data collection and analysis*: Martina Dickenmann. *Statistic and data analysis*: Mitja Nabergoj. *Data collection and analysis*: Helen Baldomero, Stavroula Masouridi‐Levrat, , Gayathri Nair, Urs Schanz, Jacob Passweg, and Alicia Rovo. *Idea of the study, data collection, analysis, interpretation, and manuscript writing*: Yves Chalandon and Ekaterina Rebmann Chigrinova.

## CONFLICTS OF INTEREST

The authors have no conflicts of interest to declare that are relevant to the content of this article. Yves Chalandon received consulting fees from MSD, Novartis, Incyte, BMS, Pfizer, Abbvie, Roche, Jazz, Gilead, Amgen, Astra‐Zeneca, and Servier; travel support from MSD, Roche, Gilead, Amgen, Incyte, Abbvie, Janssen, Astra‐Zeneca, and Jazz. Remaining authors declare they have no conflicts of interest.

## FUNDING INFORMATION

The authors did not receive support from any organization for the submitted work.

## ETHICS STATEMENT

All procedures were in accordance with the ethical standards of the respective local research committee and with the 1964 Helsinki declaration and its later amendments or comparable ethical standards.

## PATIENT CONSENT STATEMENT

Informed consent was obtained from all individual participants included in the SBST register.

## Data Availability

The data that support the findings of this study are available on request from the corresponding author. The data are not publicly available due to privacy or ethical restrictions.
